# Discrimination of thermophilic and mesophilic proteins

**DOI:** 10.1186/1472-6807-10-S1-S5

**Published:** 2010-05-17

**Authors:** Todd J Taylor, Iosif I Vaisman

**Affiliations:** 1 National Cancer Institute, Laboratory of Molecular Biology, 37 Convent Dr., MS 4264, Bethesda, MD 20892, USA; 2 Department of Bioinformatics and Computational Biology, George Mason University, 10900 University Blvd., Manassas VA 20110, USA

## Abstract

**Background:**

There is a considerable literature on the source of the thermostability of proteins from thermophilic organisms. Understanding the mechanisms for this thermostability would provide insights into proteins generally and permit the design of synthetic hyperstable biocatalysts.

**Results:**

We have systematically tested a large number of sequence and structure derived quantities for their ability to discriminate thermostable proteins from their non-thermostable orthologs using sets of mesophile-thermophile ortholog pairs. Most of the quantities tested correspond to properties previously reported to be associated with thermostability. Many of the structure related properties were derived from the Delaunay tessellation of protein structures.

**Conclusions:**

Carefully selected sequence based indices discriminate better than purely structure based indices. Combined sequence and structure based indices improve performance somewhat further. Based on our analysis, the strongest contributors to thermostability are an increase in ion pairs on the protein surface and a more strongly hydrophobic interior.

## Background

### Mesophiles, thermophiles, and hyperthermophiles

     Organisms that thrive at very high temperatures have been actively studied since the discovery of Thermus aquaticus in the hot springs of Yellowstone in the 1960’s [1]. Heat tolerant organisms are often separated into two classes: thermophiles, which have optimum growth temperatures (OGT) in the range 45-80 °C, and hyperthermophiles with OGTs above 80 °C. Mesophilic organisms are defined as those with OGT’s between 15 °C and 45 °C, while psychrophiles, which we do not address here, have OGT’s no greater than 15 °C. Sometimes the break points between these classes are assigned slightly differently. Hyperthermophiles come mostly from the kingdom Archea, but there are two genera of hyperthermophilic Eubacteria, namely Thermotogales and Aquifex. Thermophiles are more phylogenetically diverse and include Eubacteria, Archea, and some fungi.

     In addition to providing insights into the principles of protein folding and stability, understanding what makes some proteins more thermostable than others is of practical interest. Thermophilic proteins are more resistant to proteolysis and chemical denaturation, hence there is interest in engineering hyperstable biocatalysts relying on the same mechanisms that nature uses [2-4]. Thermophilic polymerases, proteases, and xylanases already have industrial applications [4,5].

### The physical basis of thermophilic protein stability

     The search for the physical basis of thermostability in proteins goes back 30 years to the work of Perutz [6].  Since then, a great many papers have been written on the subject. Some of the proposed mechanisms/indicators of increased thermostability include: a more highly hydrophobic core [7,8], tighter packing or compactness [9], deleted or shortened loops [10,11], greater rigidity [3,12,13] (for example through increased Proline content in loops), higher secondary structure content [14], greater polar surface area [15], fewer and/or smaller voids [14,16], smaller surface area to volume ratio [17], fewer thermolabile residues [16,18], increased hydrogen bonding [15], higher isoelectric point [19], and more salt bridges/ion pairs and networks of salt bridges [6,20-25].   

     Statistically significant changes in sequence composition between mesophilic and thermophilic proteins have been reported. The amino acids Asn, Gln, Met, and Cys are thermolabile—they are not stable at high temperatures and tend to undergo deamidation (Asn and Gln) or oxidation (Met and Cys) [22]. These amino acids are less common in thermophilic proteins and the thermolabile residues that do occur are usually buried [16]. Ile is preferred to Leu in hydrophobic regions of the structure because the side chain carbons can exist in all three χ rotameric states compared to only two for Leu which can result in tighter side chain packing [16]. Farias and Bonato [26] have reported that Gly, Lys, Tyr, and Ile are preferred in thermophilic organisms while Gln, His, Ala, and Cys are preferred in mesophiles. Camillau and Claverie [27] have reported that thermophilic proteins have less Gln, Ala, and His on their surfaces than mesophilic proteins do and more charged residues on their surfaces, particularly Lys and Glu. Haney et al. [28] have compared 115 proteins from Methanococcus jannaschii to mesophilic proteins from other Methanococcus species and found that the frequencies of Ile, Glu, Arg, Lys, Pro, and Tyr are significantly greater in the thermophile and the frequencies of Gly, Met, Gln, Thr, Asn, and Ser are smaller.

     More ion pairs have been strongly and consistently linked with thermostability in the literature. Water has a dielectric constant of about 80 at 0°C, which drops to 55 at 100°C and is lower still at the extreme pressures near hydrothermal vents in the deep sea where some hyperthermophilic organisms live. A lower dielectric constant makes electrostatic interactions stronger and therefore ion pairs should have a greater stabilizing effect at high temperatures and pressures [21,29].

     Evidence for some of these proposed mechanisms/indicators is equivocal. For instance, Karshikoff and Ladenstein found no significant difference in packing between thermophilic and mesophilic proteins [30] and salt bridges in a protein core have been reported to be destabilizing [31,32]. Das and Gerstein [33] have reported that the lengths of proteins from the eubacterium Aquifex aeolicus are greater than those of archeal hyperthermophilic orthologs and therefore hyperthermophilic proteins may be shorter than their mesophilic counterparts simply because most hyperthermophiles are archeal, not necessarily because shorter loops promote enhanced thermostability. Querol et al. [34] surveyed 122 references for 195 single point mutants which have been unambiguously linked to greater thermostability and found that greater rigidity, as measured by crystallographic B-factors, is not a good indicator of thermostability.

**Figure 1:**
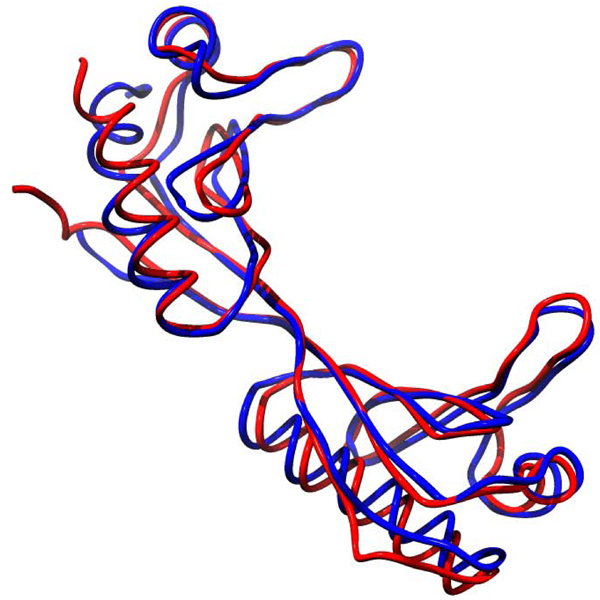
The CE structural alignment of 1aisA, a TATA-box-binding protein from the extreme thermophile Pyrococcus woesi , in red and 1ytbA, a TATA-box-binding protein from the mesophile Saccharomyces cerevisiae, in blue. The rmsd is 2Å and sequence identity is 40%. Clearly these are very similar structures.

     The overall view one comes away with from this body of work is that increased thermostability is due to relatively subtle differences in sequence and structure so that thermophilic and mesophilic orthologs are quite similar proteins (Fig. 1). They share the same catalytic mechanisms [35], although activity is typically lower at low temperatures for thermophilic enzymes [3]. The structures are similar, and sequence identity is usually, but not always, reasonably high.

### Discrimination of thermophiles and mesophiles

     Liang et al. have studied the proteomes of 15 thermophiles and 74 mesophiles using the tendencies of residue pairs separated by no more than 20 in primary sequence to occur together to discriminate mesophilic from thermophilic proteomes [36]. Farias and Bonato [26] have devised a sequence composition based index capable of correctly classifying organisms. The index r_i_ is characteristic of a single protein and is defined as r_i_=(E+K)/(Q+H) where E, K, Q, and H are the percent compositions of these amino acid types in protein i. Those authors took the average of r_i_ over all the proteins in an organism to give an average r that, without exception, fell in different ranges for the mesophiles (r < 2.5), thermophiles (3.2 < r < 4.6), and hyperthermophiles (r > 4.5) in their test set. Further, they showed that r is high in chaperonins (heat shock proteins) in both mesophiles and thermophiles [26] thereby concluding that the sequence signal is indicative of thermostability and not a phylogenetic artifact. Similarly, Claverie and colleagues [27,37] have devised the CvP bias (charged versus polar), defined as (D+E+K+R)-(N+Q+S+T), the sum of amino acid compositions, that they have also used to classify an organism as mesophilic, thermophilic, or hyperthermophilic by computing the average CvP for all the proteins from that organism. Zeldovich et al. [38] have reported a sequence composition based index defined as I+V+Y+W+R+E+L (abbreviated IVYWREL) that was an extremely good predictor of thermostability when averaged over whole proteomes, and even for just the membrane proteins from these proteomes. IVYWREL, again when averaged over proteomes, also correlates very well with OGT. None of these authors claim that their indices work well at discriminating individual pairs of thermophilic and mesophilic orthologs, however it is natural to ask if they can, and we will test this question here. Glyakina et al. have done one of the few large scale structure based discrimination studies [39].

### Delaunay tessellation of protein structures and quantities derived from it

**Figure 2:**
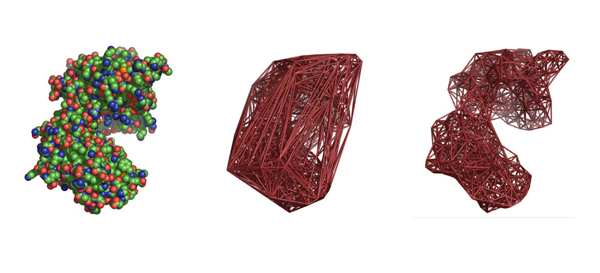
The all atom Van Der Waals spacefill representation (left) of phosphoglycerate kinase (PDB code 16pk), the Delaunay tessellation of 16pk with no simplex edge length cutoff (middle), and a view of the tessellation with a 10 Å cutoff (right). Notice that the surface of the tessellation with a cutoff corresponds more closely to that of the real molecule.

     We will refer to some Delaunay tessellation based descriptors of protein structure, so a brief introduction is in order. Delaunay tessellation, a technique for decomposing a point set into non-overlapping tetrahedral subsets, has proven very versatile in the analysis of protein structures [40-57]. With this technique, the protein is abstracted to a set of points, here the α-carbons.  These points are joined by edges in a unique way to form a set of non-overlapping, irregular, space-filling tetrahedra also known as Delaunay simplices (Fig 2) [58]. Residues joined by a Delaunay simplex edge are natural nearest neighbors in a well defined sense [58]. The analysis of statistical characteristics of the tessellation of proteins has been used in fold recognition [42-44], for structure alignment and comparison [45,46,56,59], as a way to identify cavities in the surface of a protein that could be potential binding pockets [48], to predict the stability and activity effects of point mutations [49,50], to define structural motifs [51-54], and to assign secondary structure [55]. 

     A Delaunay tessellation derived four body statistical contact pseudo-potential has been reported previously [42,43] which has been shown to contain more information than pairwise contact potentials [60]. Under this pseudo-potential, the score of some particular amino acid quadruplet (i, j, k, l), which corresponds to the residues at the vertices of a Delaunay simplex, is defined as:                     

                           (1)

where: f_ijkl_ is the observed frequency of simplices with amino acid types i, j, k, and l at their vertices in a large non-redundant training set S; a_i_, a_j,_ a_k,_ and a_l_ are the observed frequencies of the individual amino acid types in S; and c is a combinatorial factor. Variations of this potential have been successfully applied to fold recognition [43,50] and the analysis of protein stability [44] and activity [49]. We will denote by Q the sum of the log-likelihoods q_ijkl_ from the residue quadruplets corresponding to all Delaunay simplices in the tessellation of a protein structure.

     The radius of the sphere circumscribing a Delaunay tetrahedron gives a measure of its eccentricity. The small, nearly equilateral tetrahedra in the interior of the tessellation have small circumsphere radii, on the order of the size of the simplices. Radically skewed, nearly coplanar tetrahedra on the surface of the tessellated protein, on the other hand, can have circumsphere radii orders of magnitude larger than the diameter of the molecule. The *tetrahedrality* T is another measure of simplex eccentricity. Denote the length of the six edges of a simplex as L_1_ - L_6_ . The tetrahedrality is then defined by:

        (2)

### Protein contact graphs

     A few protein structure descriptors we will use are based on molecule contact graphs, so a brief introduction to this is also in order. The residues in contact with one another in a protein can be thought of as a graph or network (Fig. 2) and analyzed using techniques from elementary graph theory and the theory of complex networks. In the literature, residues are typically defined to be in contact by a simple proximity cutoff, but in this work, graph nodes correspond to residues and graph edges join nodes when the corresponding residues are joined by a Delaunay simplex, shorter than some fixed cutoff, in the tessellation of the protein structure.

     Several contact network derived quantities have been used before to analyze protein structures [61,62]. The *degree* k of a node in an undirected graph is the number of edges impinging on it. The average degree over all nodes in the contact graph will be referred to as the *coordination number*. A *minimum path* between nodes i and j is one for which the sum of weights of the edges along the path is smallest from among all possible paths. The *minimum path length* L_ij_ between nodes i and j is the sum of the weights along a minimum path. In our case here, edges have weight one, and a minimum path is one for which the fewest edges are traversed. The *characteristic path length* L of a network is the average of the minimum paths between all node pairs i, j where i ≠ j. In general, there will be many paths between distinct nodes i and j that have the minimum path length. Some classes of networks have the *clustering property*, which means that two nodes which are both joined by edges to a third, are more likely to also be joined to each other than are two nodes picked at random [63]. In such networks, there are well defined neighborhoods with subsets of nodes tending to be connected to each other and tending not to be connected to nodes in other neighborhoods. The clustering property is measured by the *clustering coefficient* of a node C_n_ is the number of actual edges E_n_ between neighbors of node n divided by the number of possible connections between those neighbors: C_n_ = 2E_n_/(k(k-1)), where k is the degree of node n. The clustering coefficient C for the entire network is the average of all the C_n_.

## Results

### Sequences differences

     Linear least square best fit lines of number of residues in hyperthermophilic (N_h_) or thermophilic proteins (N_t_) to the number of residues in their mesophilic counterparts (N_m_) are: N_h_=0.94 N_m_ + 5.00 and N_t_=0.97 N_m_ + 5.15 where there are 122 pairs in the *hyperthermophile pairs* and 127 in *thermophile pairs* (see Additional files 1 and 2 for lists). Our data show that thermophilic proteins are usually somewhat shorter than their mesophilic counterparts, and hyperthermophilic proteins are shorter still. This observation is in line with the results of Eisenberg [11].

     Tables 1 and 2 show the amino acid composition for a large nonredundant set of thermophilic, hyperthermophilic, and mesophilic proteins. T-tests were conducted to see if the average composition was different for the hyperthermophiles, thermophiles, and a control set of mesophiles. Table 1 is in broad agreement with previously published results: there are more charged residues in thermophilic proteins and fewer polar and thermolabile residues. Arg is preferred in thermophilic proteins but Lys is preferred in hyperthermophilic proteins [14].

**Table 1:** t-tests of sequence percent compositions of mesophilc, thermophilic, and hyperthermophilic proteins.

	** A**	**C**	**D**	**E**	**F**	**G**	**H**	**I**	**K**	**L**
mean_comp_meso:	8.26	1.92	5.80	6.53	3.85	7.29	2.29	5.48	6.22	8.86
sd_comp_meso:	4.31	3.05	2.22	3.02	1.89	3.08	1.53	2.52	3.38	3.52
										
mean_comp_therm:	10.05	0.78	5.13	8.32	3.62	8.30	2.15	4.80	4.77	10.26
sd_comp_therm:	3.48	1.23	2.29	2.87	1.45	2.12	1.18	3.04	2.29	3.87
t_therm_wrt_meso:	6.03	-9.08	-3.57	7.49	-1.83	5.42	-1.35	-2.72	-7.21	4.40
										
mean_comp_hyper:	7.26	0.78	5.20	10.05	4.07	7.17	1.64	7.73	8.50	9.10
sd_comp_hyper:	2.95	1.05	1.76	2.51	1.64	2.23	1.05	2.44	2.60	2.39
t_hyper_wrt_meso:	-4.09	-10.19	-4.17	17.47	1.72	-0.65	-7.37	11.72	10.74	1.21
										
t_therm_wrt_hyp:	7.99	0.00	-0.32	-5.93	-2.71	4.83	4.22	-9.80	-14.19	3.30
										
	**M**	**N**	**P**	**Q**	**R**	**S**	**T**	**V**	**W**	**Y**
mean_comp_meso:	2.17	4.51	4.52	4.04	4.79	6.07	5.62	6.90	1.43	3.44
sd_comp_meso:	1.58	2.30	2.91	2.07	2.62	2.61	2.51	2.62	1.25	1.95
										
mean_comp_therm:	1.87	3.19	5.43	2.69	6.82	4.07	4.74	8.42	1.31	3.30
sd_comp_therm:	1.11	2.29	2.05	1.59	2.81	2.13	2.34	2.36	1.16	1.71
t_therm_wrt_meso:	-3.14	-6.94	5.10	-9.87	8.77	-11.07	-4.52	7.65	-1.20	-1.00
										
mean_comp_hyper:	2.11	3.49	4.02	1.80	5.56	4.51	4.00	8.44	1.02	3.56
sd_comp_hyper:	1.14	1.62	1.57	1.16	2.27	1.72	1.48	2.28	1.07	1.71
t_hyper_wrt_meso:	-0.59	-7.65	-3.60	-22.12	4.23	-10.82	-12.71	8.45	-4.78	0.87
										
t_therm_wrt_hyp:	-1.98	-1.39	7.11	5.88	4.55	-2.10	3.46	-0.08	2.41	-1.41

t-tests of sequence percent compositions of mesophilc, thermophilic, and hyperthermophilic proteins Amino acids significantly over-represented in hyperthermophiles with respect to mesophiles are: Glu, Ile, Lys, Arg, and Val. Amino acids significantly under-represented in hyperthermophiles with respect to mesophiles are: Ala, Cys, Asp, His, Asn, Pro, Gln, Ser, Thr, and Trp. Amino acids significantly over-represented in thermophiles with respect to mesophiles are: Ala, Glu, Gly, Leu, Pro, Arg, and Val. Amino acids significantly under-represented in thermophiles with respect to mesophiles are: Cys, Asp, Lys, Met, Asn, Gln, Ser, and Thr. Amino acids significantly over-represented in hyperthermophiles with respect to thermophiles are: Glu, Ile, and Lys. Amino acids significantly under-represented in hyperthermophiles with respect to thermophiles are: Ala, Gly, His, Leu, Pro, Gln, Arg, and Thr. These statistics were tabulated from nonredundant sets of 184 hyperthermophilic structures (45419 residues), 162 thermophilic structures (41470 residues), and 1262 mesophilic structures (269799 residues).

**Table 2:** Statistical significance of fractions in Table 1.

Fraction	P for thermophile pairs	P for hyperthermophile pairs
0.50	1.000	1.000
0.55	0.297	0.319
0.60	0.0328	0.0369
0.65	6.85 x 10^-4^	0.00143
0.70	6.97 x 10^-6^	1.65 x 10^-5^
0.75	1.94 x 10^-8^	4.99 x 10^-8^
0.80	3.12 x 10^-12^	8.34 x 10^-12^
0.85	2.66 x 10^-16^	6.78 x 10^-16^
0.90	<2.20 x 10^-16^	<2.20 x 10^-16^
0.95	<2.20 x 10^-16^	<2.20 x 10^-16^

Statistical significance of the fractions in Table 1 given by a two-sided binomial test.  Fractions below ~0.6-0.65 should not be considered signficant.

### Results of discrimination experiment

     From Table 3 it can be seen that simplex geometry based indices are generally poor discriminators as are contact network based indices. Some compactness-based indices are good discriminators, for example Delaunay area/volume, a measure of general compactness, and van der Waals volume/Delaunay volume, a measure of void space. Secondary structure content, rigidity as measured by the mean B-factor, and sequence length are not very good discriminators. Sequence composition based indices, particularly IVYWREL and CvP are very good discriminators. Delaunay derived combined sequence-structure indices are very good discriminators as well, for example the 4-body potentials and the counts of over-represented residue quadruplets. Interestingly, even though the 4-body Delaunay threading potential works well as a discriminator, this is apparently not true for threading potentials in general. We have tested the ProsaII potential of Sippl et al [64], and found it to be a poor discriminator (Table 3).

**Table 3:** Discriminatory power of  structure and sequence derived quantities

Numerical index	Thermophile (127 pairs)	Hyperthermophile (122 pairs)
**Contact Network Derived Quantities**		
coordination number (no cutoff)	0.559	0.689
clustering coefficient (no cutoff)	0.551	0.672
characteristic path (no cutoff)	0.520	0.631
**Combined Sequence and Structure Including Threading Potentials**		
total count 400 over-rep quads/residue	0.850	0.943
4-body potential/residue (20Å cutoff)	0.858	0.844
4-body potential/residue (no cutoff)	0.843	0.852
4-body potential/res (hyper only, no cutoff)	-----	0.820
4-body potential/res (meso only, no cutoff)	0.732	0.803
4-body potential/res (thermo only,no cutoff)	0.866	-----
ProsaII combined score	0.554	0.693
**Delaunay Simplex Geometry**		
median circumsphere radius(no cutoff)	0.701	0.639
mean tetrahedrality (no cutoff)	0.598	0.574
number simplices/residue (10Å cutoff)	0.528	0.557
number simplices/residue (no cutoff)	0.567	0.697
**Volume/Surface Area/Compactness**		
Naccess solvent accessible area	0.567	0.598
Delaunay surface area (no cutoff)	0.606	0.669
van der Waals area	0.559	0.549
Delaunay volume (no cutoff)	0.598	0.701
Van der Waals volume	0.528	0.598
Delaunay area/volume (10Å cutoff)	0.583	0.549
Delaunay area/volume (no cutoff)	0.669	0.803
van der Waals area/volume	0.512	0.557
packing density	0.543	0.549
van der Waals volume/Delaunay volume	0.685	0.779
**Rigidity**		
mean B-factor	0.661	0.533
**Secondary Structure**		
secondary structure content (H+E 3 state DSSP)	0.614	0.689
**Sequence Length**		
number of residues	0.528	0.672
**Sequence Composition**		
total Kyte-Doolittle hydrophobicity	0.575	0.549
sd Kyte-Doolittle hydrophobicity	0.677	0.836
CvP bias	0.803	0.918
(E+K)/(Q+H)	0.591	0.861
IVYWREL	0.827	0.926

A table showing the discriminatory power of sequence and structure based indices-the fraction of thermophile/mesophile pairs for which the quantity was systematically higher or lower by any amount. The contact network quantities are described in the introduction. The four body threading contact potentials are described in [1]. The cutoff indicates that simplices with at least one edge longer than the cutoff were omitted when frequencies are tallied during the calculation of the potential. "Hyper only" indicates that the potential was trained only on chains from  hyperthermophilic organisms. The Delaunay simplex geometry indices are discussed in the introduction.  The volume and surface area criteria are fairly self-explanatory except, perhaps, for packing density that is defined here as the ratio of the van der Waals volume of the protein divided by the all atom Voronoi volume. The sequence composition based indices CvP, (E+K)/(Q+H), and IVYWREL are described in the introduction.

     The best discriminatory indices we tested were: one version of the 4-body Delaunay threading potential; the count of over-represented quadruplets; the ratio of Delaunay surface area to volume (for hyperthermophiles); the standard deviation of Kyte-Doolittle hydrophobicity (for hyperthermophiles); Delaunay area/volume=and van der Waals volume/Delaunay volume, particularly for hyperthermophiles; the CvP bias, and IVYWREL. Few of the tested indices (when computed for individual proteins not averaged over proteomes) correlate even moderately well with OGT. Those for which the correlation is strongest are the 4-body potentials, *overrep400*, CvP, IVYWREL, and E+K/Q+H which all have r~0.4-0.7.

     The Delaunay simplex-based descriptors (*overrep400* and the 4-body potentials) that work best for discrimination use large simplex edge length cutoffs ( >20 Å). This implies that there are important residue contacts on the surface of the proteins because that is invariably where simplices with very long edge lengths reside. This combined with threading potential data presented later leads us to believe that the presence of more charged residues on the protein surface is at least one of the things these descriptors pick up.

      Since increased hydrophobicity of the protein core has been proposed as a mechanism for thermostability, one might expect the sum S_KD_ of the Kyte-Doolittle hydrophobicities of all residues would be a good discriminator. S_KD_ is not, but the variance of the Kyte-Doolittle hydrophobicities is (Table 3). Apparently, then, the increase in core hydrophobicity is accompanied by an increase in hydrophilic residues.

### Why the Delaunay threading potential a good discriminator

     We have studied the mean contribution to the 4-body potential score Q under a 7-letter reduced alphabet to each structure in the mesophilic, thermophilic, and hyperthermophilic subsets of *521nonredundant*. The reduced alphabet was used to simplify the analysis by bringing down the possible number of residue quadruplets that can reside at the vertices of a Delaunay tetrahedron from 8855 for a 20 amino acid alphabet to 210. The reduced alphabet is D, E, K, R, I=(I,V), A=(A,F,G,L,P), N=(C,H,M,N,Q,S,T,W,Y. The four-body Delaunay threading score Q for a hyperthermophile with the average quadruplet composition is 36.3, for a thermophile Q is 28.2, and for a mesophile Q is 17.6. For hyperthermophiles, the ΔQ (with respect to mesophiles) attributable to quadruplets with at least two hydrophobic residues is +11.07, for quadruplets with at least two charged residues it is +12.78, and for quadruplets with at least two polar residues it is –2.53. For thermophilic quadruplets the ΔQ figures are: at least two hydrophobic residues +5.99, at least two charged residues +4.55, and at least two polar residues –0.34. We see ,therefore, that the 4-body potential goes up for (hyper)thermophiles both due to associations between charged residues but also due to quadruplets of more highly hydrophobic residues. The increase in charged residues produces a larger change than the stronger hydrophobics with hyperthermophiles, but the situation is reversed in thermophiles.

## Conclusions

     It is possible to accurately discriminate (hyper) thermophilic proteins from their mesophilic counterparts based on sequence and structural properties. Sequence based indices used to discriminate entire proteomes also work well on individual thermophile/mesophile ortholog pairs. Purely structure-based indices are, generally speaking, poor discriminators. Combined sequence structure indices like the threading potential are somewhat better than sequence alone.

     The primary factors differentiating thermophilic from mesophilic proteins according to our analysis are surface ion pairs and more strongly hydrophobic core residues. The conclusion of previous authors that the basis for the thermostability of thermophiles and hyper-thermophiles is somewhat different is also borne out here (e.g. the preference of thermophiles for Arg and of hyperthermophiles for Lys).

     Extensions of this work currently underway include compiling larger test sets and breaking them down by kingdom of origin as well as OGT. Heat shock proteins should be compared to regular proteins from the same organism and non-thermophilic archeal proteins should be compared to orthologs from thermophiles. Proteins from psychrophiles should be analyzed too. It would be a small step to use more sophisticated pattern recognition methods to discriminate or classify based on multiple indices.

     Finally, it may be possible to design a thermostable protein from a non-thermostable one by an adaptive walk in sequence space, threading the altered sequences onto the structure of the non-thermostable protein, such that one or more of the good discriminators described here always increases.

## Methods

### Assembly of the test sets

     In this paper, we have addressed *the discrimination problem* where given sequences or structures from a mesophilic protein and a thermophilic or hyperthermophilic counterpart, the objective is to determine which is which. This was done by assembling a large set of thermophilic protein chains from the PDB and their corresponding mesophilic analogs and another large non-redundant set of hyperthermophilic PDB protein chains along with their mesophilic analogs. They will be referred to as the *pairs* sets*: pairs-thermophile* and* pairs-hyperthermophile*. We have computed several structure and sequence based numerical indices, based on the quantities that other authors have reported are associated with thermostability, and tested their ability, individually, to successfully discriminate between the thermophile/mesophile pairs. One could apply more sophisticated classification or regression techniques to a combination of these quantities, but for now we have opted for a very simple test of each quantity in isolation in order to verify if it is indeed consistently associated with increased thermostability.

     The *pairs sets* were constructed to contain pairs of high quality x-ray structures with high structural and functional similarity that differ only in that one is mesophilic and the other thermophilic or hyperthermophilic. They were assembled by compiling all PDB x-ray structures from a large list of organisms categorized as mesophile, thermophile or hyperthermophile using OGT’s obtained from the ATCC website (http://www.atcc.org/common/catalog/bacteria/bacteriaIndex.cfm). All structures with missing Cα coordinates, insertion codes, or alternate atoms were then eliminated. The resulting two lists of thermophilic and hyperthermophilic proteins were submitted separately to PISCES[65] to generate two much smaller non-redundant sets in which all members had crystallographic resolution no greater than 2.2 Å, an R-factor no greater than 0.23, and where no pair of structures had a sequence identity greater than 30%. The members of these non-redundant sets of chains from thermophilic organisms were each then submitted to structure comparison and alignment servers (CE[66] , SSM [67], DALI[68], VAST[69]) to obtain mesophilic structure neighbors with rmsd no greater than about 4 Å with respect to the thermophile where the structural alignment included ~80% or more of each structure, and where the thermophile and mesophile had identical or close EC numbers or functional annotation. In some cases, more than one mesophile per thermophilic protein was kept. When multiple mesophilic analogs to a single thermophilic protein were included in the *pairs* sets, no restriction was placed on their similarity with respect to each other except that the sequences not be identical and that they come from different organisms. Lists of the resulting structure pairs and structure alignment data can be found among the supplementary material. The structural alignment data in these tables were computed using CE [66].

     To train threading potentials and compute sequence composition biases, we compiled a second set of PDB x-ray structures (*521nonredundant*), larger and more representative than the *pairs* sets. The set contained 175 mesophilic, 162 thermophilic, and 184 hyperthermophilic protein structures, none of which was in either *pairs* set. As with the *pairs* sets, *521nonredundant* was assembled with the help of PISCES [65] All members had crystallographic resolution no greater than 2.2 Å, an r-factor no greater than 0.23, and no pair of structures had a sequence identity greater than 66%. The pairwise similarity threshold was set higher for this set than *pairs* in order to allow the possibility that it could contain mesophilic and thermophilic orthologs, however a lower similarity threshold would have made little difference—a 30% similarity cutoff would have eliminated only 41 structures.

### Numerical discriminators tested

     We have computed several structure and sequence based numerical indices to see if they can successfully discriminate between the related mesophilic and (hyper)thermophilic proteins in *the pairs* sets (Table 3). Among the quantities tested for discriminatory power were: the three contact-network derived quantities described in the introduction (coordination number, characteristic path length, and clustering coefficien), the ratio (E+K)/(Q+R) of Farias and Bonato [26], the CvP bias defined earlier [37], the sum and standard deviation of the Kyte-Doolittle hydrophobicities of all residues in the protein, several four-body Delaunay tessellation based threading Q-scores [42], the ProsaII two-body distance dependent threading score [64], the median circumsphere radius, the mean tetrahedrality, the surface area to volume ratio (both Delaunay and van der Waals), and the number of Delaunay simplices per residue. We have also tested the packing density defined as the van der Waals volume of the protein divided by the all-atom Voronoi volume.  The algorithm of Gavezotti [70] was used to calculate van der Waals volumes and the Geometry Code Library 2.0 of Tsai et al. [71] to calculate Voronoi volumes.

      We have defined discriminatory power of a given numerical index as simply the fraction f of (hyper)thermophile-mesophile pairs from a *pairs* set where that index is systematically larger or smaller for the (hyper)thermophilic protein, by any amount. For example, 86.1% of the time for structure pairs in the *pairs hyperthermophile* set, the ratio (E+K)/(Q+H) is greater for the hyperthermophilic than for the mesophilic protein, and so the discriminatory power is 0.861.

     Analysis of the residue quadruplets occupying the vertices of the tetrahedra of tessellated hyperthermophilic and thermophilic protein structures shows that some are heavily over-represented (e.g. EEEK, AEER) or under-represented (e.g. ANQV, AELR) in (hyper)thermophilic proteins with respect to mesophilic (Table 4). Another discriminator index we have tested on the *pairs* sets, therefore, is the total count in a query protein of the top 400 most over-represented simplices in hyperthermophilic or thermophilic structures divided by the number of residues. These two indices are the most powerful discriminators of all those we tested. We will abbreviate them as *overrep400-thermophile* and *overrep400-hyperthermophile*.

**Table 4:** Highly over-represented and highly under-represented residue quadruplets at the vertices of tessellated thermostable proteins and the factors by which they differ with respect to mesophiles.

hyperthermophiles						thermophiles				
**over-represented**			**under-represented**			**over-represented**			**under-represented**	

**quad**	**factor**		**quad**	**factor**		**quad**	**factor**		**quad**	**factor**

EEEE	7.473		PQRT	0.303		EEER	6.008		AIQS	0.380
EEEK	7.332		GGNQ	0.302		ELWW	5.520		DLQS	0.378
EEER	7.048		FLLQ	0.301		RRRV	5.222		KLST	0.377
MRRR	6.490		AQVY	0.301		AEER	5.106		ILNQ	0.373
EEKR	5.654		AAAN	0.299		EEPR	5.087		ENQS	0.370
IRRR	5.605		AAEQ	0.298		EERR	4.801		KKLS	0.370
EEEF	5.597		DSTT	0.296		AERR	4.538		KNQR	0.368
EEKK	5.282		ADDT	0.296		RRRY	4.508		FKQS	0.368
EEEV	4.936		AENQ	0.296		EEEE	4.508		LNQS	0.367
EEIK	4.889		NSTT	0.295		ERRR	4.458		EFNS	0.366
EIKK	4.881		ALPQ	0.292		IRRR	4.186		DILQ	0.365
EEIV	4.346		ADQR	0.292		EEGP	4.177		LMSY	0.364
EIKR	4.332		ANRT	0.292		EEGR	4.113		KLNS	0.362
EKRR	4.256		DNQV	0.292		ERRV	4.104		DDQS	0.361
IKRR	4.228		FGLQ	0.291		EGPR	4.092		LNSS	0.361
EEKV	4.225		AQST	0.291		EERW	4.015		EHIK	0.360
EEIR	4.169		ANTY	0.290		GPRR	3.963		KKST	0.358
EEEN	4.107		INSY	0.290		EEPP	3.951		GKSS	0.356
EEEY	4.092		DNQT	0.289		EERV	3.904		CGLL	0.354
KRRR	4.085		ANQV	0.289		AELR	3.792		LNQT	0.353

## Competing interests

     The authors declare that they have no competing interests.

## Authors' contributions

     IIV originated the analysis of protein structure using Delaunay tessellation and the four-body pseudo potential. TJT assembled the test sets, determined which quantities to test, and wrote scripts to compute tessellation and sequence derived quantities. TJT drafted the paper, which was approved by IIV.

## Supplementary Material

Additional file 1 pairs_hyperthermophiles.txt

Additional file 2 pairs_thermophiles.txt
